# Parental involvement and children’s cognitive and non-cognitive development: evidence from China

**DOI:** 10.3389/fpsyg.2026.1748832

**Published:** 2026-02-17

**Authors:** Hengli Wang, Ziyan Zhan, Zuojunli Zhang

**Affiliations:** 1 Wuhan University of Cyber Security Preparatory Office, Wuhan, China; 2 Independent Researcher, Wuhan, China

**Keywords:** cognitive development, family environment, learning engagement, non-cognitive skills, parental involvement

## Abstract

The development of children’s cognitive and non-cognitive abilities is a central concern in education and human capital research. Using nationally representative data from the China Education Panel Survey (CEPS), this study examines how parental involvement is associated with children’s cognitive and non-cognitive development in China. We focus on three dimensions of parental involvement—communication frequency, shared activities, and emotional closeness—and estimate their relationships with children’s cognitive skills and key non-cognitive traits, including emotional stability, conscientiousness, and agreeableness. The results show that higher levels of parental involvement are positively associated with both cognitive and non-cognitive outcomes. These associations remain robust across alternative specifications and quantile regressions. Further analyses reveal meaningful heterogeneity by gender, while differences by household registration status and boarding arrangements are less systematic. To explore the mechanisms underlying these relationships, the study conducts mediation analyses focusing on learning engagement and children’s confidence. The findings show that learning engagement serves as an important behavioral channel linking parental involvement to cognitive development, while both learning engagement and confidence play significant roles in shaping non-cognitive outcomes. Together, these results highlight that parental involvement influences children’s development through both behavioral and psychological pathways. Overall, the findings provide additional evidence on the role of parental involvement in children’s multidimensional development within China’s institutional and cultural setting and suggest that policies aimed at supporting parental engagement may contribute to improved child development outcomes.

## 1 Introduction

Children’s cognitive and non-cognitive development is shaped by both formal schooling and also by the informal learning environment within the family. A growing body of research highlights that parental involvement plays a crucial role in shaping children’s academic performance, socio-emotional skills, and long-term human capital accumulation (Joy et al., 2021). Through daily communication, shared activities, and emotional interaction, parents provide both the cognitive stimulation and the socio-emotional support that influence children’s learning trajectories.

Parental involvement refers to a set of concrete behaviors through which parents interact with their children in daily life. While parent–child closeness reflects emotional bonds, parental involvement captures concrete and observable behaviors through which parents participate in children’s daily activities, structure informal learning environments, and transmit educational values. As such, parental involvement functions as an independent behavioral input and a key channel through which parent–child relationships affect children’s developmental outcomes.

In recent decades, rapid socioeconomic transformation has profoundly altered family structures and parenting practices globally. Increased work intensity, urbanization, and increasing educational competition have made parenting more demanding and time-consuming (Begall and Mills, 2011; Hook et al., 2022). Against this backdrop, parental involvement has become an increasingly critical factor in children’s development, particularly in terms of shaping both cognitive and non-cognitive skills (Cheung et al., 2022). However, the nature and effectiveness of parental involvement may vary substantially across institutional and cultural contexts, and thus, there is a need for context-specific empirical analysis.

China provides a particularly informative setting for examining the above issues. Chinese parenting is deeply influenced and shaped by Confucian family norms, strong intergenerational expectations, and an examination-oriented education system. Parents are often heavily involved in their children’s education, with a focus on academic supervision, structured learning activities, and performance outcomes. Simultaneously, institutional arrangements such as the household registration (hukou) system and boarding school practices create unequal opportunities for daily parent–child interaction. These features distinguish China from many Western societies, and they amplify both the benefits and inequalities associated with parental involvement.

Existing literature has examined a range of family-related factors affecting children’s development. Examples include parental education, maternal employment, family income, and policy environments (Bose and Chatterjee, 2024; De Hoop et al., 2018; Menashe and Atzaba-Poria, 2016; Zhang et al., 2023). While these studies have provided important insights, relatively less attention has been paid to parents’ time-based and interaction-based involvement in children’s everyday learning. In particular, there has been a lack of use of large-scale representative data. Moreover, prior research has often focused on cognitive outcomes. Comparatively limited investigation has been conducted into non-cognitive dimensions, such as emotional regulation, persistence, and prosocial behavior. Empirical evidence on the mechanisms that link parental background, parental involvement, and children’s development also remains fragmented.

This study contributes to the existing literature by examining how parental involvement is associated with children’s cognitive and non-cognitive abilities in China. Using nationally representative data from the China Education Panel Survey (CEPS), the analysis focuses on three dimensions of parental involvement—communication frequency, shared activities, and emotional closeness—and explores their relationships with children’s developmental outcomes. In addition to baseline regressions and robustness checks, the study conducts heterogeneity analyses and mediation tests to explore both differential effects and underlying mechanisms.

This study makes three main contributions. First, using large-scale representative data, it provides comprehensive evidence on how different dimensions of parental involvement shape children’s cognitive and non-cognitive development. Second, by employing interaction-based tests, it identifies meaningful heterogeneity across gender and institutional contexts. Third, this research advances existing research by elucidating behavioral and psychological mechanisms—specifically learning engagement and confidence—through which parental involvement influences children’s development. Thus, by situating these findings within China’s distinctive institutional and cultural context, this study contributes to both the domestic literature on family and education and the broader international discussion on parental involvement and human capital formation.

The remainder of this article is organized as follows: Section 2 reviews the relevant literature, Section 3 describes the data and research design, Section 4 presents the empirical results, and Section 5 concludes with key findings, policy implications, and directions for future research.

## 2 Literature review and conceptual framework

### 2.1 Parental involvement and children’s human capital development

A substantial body of literature has documented the important role of family inputs in shaping children’s human capital development, particularly with respect to education, health, and socio-emotional skills. From the perspective of human capital theory, children’s knowledge, abilities, and health constitute key productive assets that affect their future economic opportunities (Bandelj and Spiegel, 2023). Within this framework, parental involvement has been widely recognized as a central mechanism through which families influence children’s developmental trajectories. Previous studies show that family environments, parenting practices, and parents’ socioeconomic characteristics significantly shape how children’s human capital is accumulated (Cheung et al., 2022; Wu and Zhang, 2024; Zhang et al., 2020; Zhang and Wang, 2022).

A large empirical literature emphasizes the crucial role of parental involvement in children’s cognitive development across different cultural and socioeconomic contexts (Guo and Chiu, 2023; Wu and Zhang, 2024). More specifically, parental engagement in children’s educational activities has been shown to be particularly important for the development of foundational academic skills, such as language and mathematics (Menashe and Atzaba-Poria, 2016; Skwarchuk et al., 2014; Zhang et al., 2020; Zhang and Wang, 2022). Parents with higher levels of education tend to engage more intensively in their children’s learning processes by providing cognitively stimulating interactions and structured home learning environments, thereby enhancing academic performance and cognitive skills (Guo and Chiu, 2023; Wu and Zhang, 2024). Using evidence from early childhood education, Miguel et al. (2013) document a strong positive association between parental involvement and children’s later academic achievement and career outcomes. Together, these studies underscore that parental involvement constitutes an important family input contributing to children’s early human capital formation, particularly in contexts characterized by intense educational competition and substantial parental investment.

Beyond cognitive outcomes, parental involvement also plays an important role in shaping children’s non-cognitive abilities. Parental engagement characterized by emotional support, communication, and shared activities contributes to children’s emotional stability (Midouhas et al., 2014), persistence, and social skills—traits that are increasingly recognized as essential components of human capital (Valle et al., 2016). Empirical evidence suggests that parents’ daily emotional engagement is strongly associated with improved academic performance and enhanced social interactions (Li and Guo, 2023), while parental care and support promote children’s broader socio-emotional skills (Xu et al., 2018). Moreover, authoritative parenting styles have been shown to foster self-regulation, whereas indulgent or neglectful parenting undermines emotional development (Zeng et al., 2024; Shorer et al., 2021). This effect is particularly evident in low-income families, where positive parent–child interactions help to mitigate the negative impact of limited material resources on children’s ability to accumulate human capital(Fletcher and Kim, 2019; Zhang et al., 2023).

Cross-cultural studies further indicate that the forms and intensity of parental involvement vary systematically across institutional and cultural contexts. For example, Mardis (2013) found that middle-class parents in the United States are more likely to adopt a “collaborative parenting” “approach that emphasizes personality development and social skills, whereas working-class families tend to prioritize basic material needs. Similar patterns appear globally. East Asian parents emphasize academic achievement (Chelliah et al., 2023), whereas Western contexts tend to prioritize autonomy and creativity (Yuan et al., 2016). Hess et al. (1987) found that Japanese parents engage more extensively than do American parents in academic-oriented interactions, and this contributes directly to differences in children’s cognitive outcomes. American parents tend to encourage independence and self-expression, while Chinese parents often emphasize discipline and social responsibility (Behrman et al., 2017). Despite these insights, previous research has not yet fully clarified how parental involvement simultaneously contributes to the development of cognitive and non-cognitive abilities across different cultural settings, thereby leaving space for further empirical investigation.

While this literature establishes a robust association between parental involvement and children’s cognitive and non-cognitive outcomes, it provides more limited insight into the specific behavioral mechanisms through which such effects operate and how different dimensions of parental involvement translate into children’s multidimensional development. This gap motivates the present study’s focus on identifying and empirically examining the mechanisms linking parental involvement to both cognitive and non-cognitive abilities.

### 2.2 Mechanisms linking parental involvement to cognitive and non-cognitive development

To motivate the empirical analysis, a growing literature has begun to conceptualize parental involvement as a set of behavioral processes through which family background and parenting practices are transmitted into children’s cognitive and non-cognitive outcomes. Rather than viewing parental involvement merely as a descriptive feature of the parent–child relationship (Erkenbrack, 2025), recent studies emphasize its role as an observable behavioral channel through which family resources and parental practices shape children’s human capital formation (Cotton et al., 2026).

One prominent mechanism operates through learning support and cognitive stimulation. When parents frequently communicate about school life, supervise learning routines, and engage in shared learning-related activities, children are exposed to more structured guidance and cognitively-enriching interactions (Li and Li, 2025). These inputs strengthen children’s academic engagement, persistence, and effort, thereby facilitating the accumulation of cognitive skills. In the literature, such behavioral responses are commonly conceptualized as learning engagement, which captures students’ sustained effort, commitment, and involvement in learning activities. This pathway is closely related to parental expectations and the organization of the home learning environment, which reinforce study habits and achievement-oriented behaviors (Moroni et al., 2025).

A second mechanism operates through the emotional climate and socio-emotional regulation within the family (Verhagen et al., 2025). Warm, responsive, and consistent parental involvement can enhance children’s sense of emotional security, reduce stress, and promote self-regulation and persistence (Li et al., 2025). Beyond emotional regulation, a growing body of research highlights children’s confidence or self-efficacy as a key psychological channel linking family environments to non-cognitive development. Positive parental feedback, encouragement, and support contribute to children’s beliefs about their own abilities and future prospects, which in turn shape motivation, emotional resilience, and socio-emotional skills (Kılıç et al., 2025). By helping children cope with academic pressure and setbacks, parental emotional support may thus foster confidence that complements learning-oriented engagement (Xiao et al., 2025). In contrast, limited or inconsistent parental involvement may weaken these protective functions, and this is particularly true for children who lack alternative sources of emotional support (Lau et al., 2025).

Importantly, behavioral and psychological mechanisms are not independent (Liu et al., 2025). Improvements in emotional regulation and confidence can enhance children’s attention, classroom behavior, and capacity to benefit from cognitive inputs (Xiong et al., 2021), while positive academic experiences may further strengthen self-efficacy and motivation (Djekourmane et al., 2025). Through the process of mutual reinforcement, parental involvement contributes to children’s development, not through isolated pathways, but rather through an integrated mechanism in which learning engagement and confidence jointly cognitive and non-cognitive human capital formation (Onsomu et al., 2025).

This integrated framework provides a clear conceptual foundation for the empirical analyses that follow. Guided by the existing literature, the study focuses on learning engagement as a behavioral mechanism and confidence as a psychological mechanism, and empirically examines how these two channels mediate the relationship between parental involvement and children’s cognitive and non-cognitive outcomes.

### 2.3 Research focus and mechanism selection

Building on the above literature, this study deliberately focuses on a parsimonious set of empirically observable mechanisms through which parental involvement influences children’s cognitive and non-cognitive development. Rather than attempting to exhaustively test all potential channels discussed in the literature, the analysis concentrates on two mechanisms that are both theoretically salient and empirically tractable: learning engagement and confidence.

Learning engagement is selected as a behavioral mechanism because it directly captures how parental involvement is translated into children’s sustained effort, persistence, and active participation in learning activities. Prior research consistently emphasizes that parental communication, supervision, and joint activities primarily affect children’s day-to-day learning behaviors, which are central to cognitive skill formation. Focusing on learning engagement therefore allows the analysis to examine a concrete behavioral pathway linking family inputs to cognitive outcomes.

Confidence, or self-efficacy, is chosen as a complementary psychological mechanism because it reflects children’s beliefs about their own abilities and future prospects, which are closely related to socio-emotional development. Existing studies suggest that parental encouragement and emotional support play an important role in shaping children’s motivation, resilience, and non-cognitive skills. By examining confidence, the analysis captures a distinct psychological channel through which parental involvement may influence non-cognitive outcomes beyond observable learning behaviors.

These two mechanisms are selected for three main reasons. First, they are firmly grounded in the existing theoretical and empirical literature on parental involvement and child development, as discussed in Section 2.2. Second, both mechanisms are measured directly in the CEPS data using well-defined survey items, allowing for transparent and replicable empirical implementation. Third, taken together, learning engagement and confidence enable an integrated examination of how parental involvement affects both cognitive and non-cognitive outcomes, thereby extending prior research that has often analyzed these dimensions separately.

By focusing on these two mechanisms, the study provides a coherent and targeted empirical investigation of the behavioral and psychological pathways through which parental involvement contributes to children’s multidimensional human capital development.

## 3 Data source and variables

### 3.1 Data source

The China Education Panel Survey (CEPS), with baseline data from 2013 to 2014, was used to obtain reliable data for this study. The CEPS is a nationally-representative longitudinal survey that is jointly conducted by the National Survey Research Center and Renmin University of China. The baseline survey adopts a stratified, multistage probability sampling design that is based on regional and educational characteristics.

Specifically, 28 county-level units (including counties, districts, and county-level cities) were randomly selected nationwide. Within these units, 112 middle schools and 438 classes were sampled, covering both seventh- and ninth-grade students, and all students in all of the sampled classes participated in the survey. The baseline dataset contains detailed information on the students’ academic performance, socio-emotional development, family background, and parent–child interactions. The initial sample size was approximately 20,000 students.

After excluding observations with missing values on key variables, the final analytical sample used in this study’s empirical analysis consists of approximately 18,000 to 19,000 students, depending on the precise model specification. The CEPS has been widely used in studies on children’s cognitive and non-cognitive development and provides a solid empirical foundation for examining the role of family environment and parental involvement (Li S. F. et al. 2020; Li C. K. et al. 2020; Wang et al., 2023; Tang et al., 2024). Although the data were collected in an earlier period, the CEPS remains one of the few nationally representative datasets that simultaneously captures detailed measures of parental involvement and multiple dimensions of children’s non-cognitive development.

### 3.2 Key variables

Table 1 summarizes the definitions and construction of all variables used in the empirical analysis, including dependent variables, parental involvement measures, mechanism variables, and control variables.

**Table 1:** Definitions and construction of variables.

Category	Variable	Definition
Dependent variables	Cognitive ability	Standardized composite cognitive test score constructed from CEPS baseline cognitive assessments
	Non-cognitive ability	
	Emotional stability	Composite index measuring emotional regulation and negative affect based on student questionnaire items
	Conscientiousness	Composite index measuring persistence, responsibility, and task-oriented behaviors
	Agreeableness	Composite index capturing prosocial behavior, cooperation, and interpersonal orientation
Independent variables (parental involvement)	Communication frequency	Frequency of parent–child communication regarding school life and daily experiences
	Activity frequency	Frequency of shared parent–child activities (e.g., studying, discussions, joint participation)
	Emotional closeness	Self-reported closeness between parents and children, measured on a three-point scale
Mechanism variables	Learning engagement	Composite index measuring students’ learning effort, persistence, and commitment to academic tasks
	Confidence (self-efficacy)	Index capturing students’ confidence in their academic abilities and future expectations
Control variables (individual characteristics)	Gender	Male = 1, Female = 0
	Household registration	Non-agricultural hukou = 1, agricultural hukou = 0
	Age	Student age measured in months
	Only child	Only child = 1, otherwise = 0
	Boarding status	Boarding at school = 1, otherwise = 0
	Grade	Grade 9 = 1, Grade 7 = 0
Control variables (family characteristics)	Parental relationship	Indicator of parental relationship quality (1 = good, 0 = poor)
	Parents’ education	Highest years of schooling of parents (coded from no education to postgraduate level)
	Family economic status	Self-reported household economic condition (1 = difficult, 2 = average, 3 = affluent)

#### 3.2.1 Dependent variables

In this study, children’s cognitive ability and non-cognitive ability are treated as two dependent variables, each representing key components of human capital. To be specific, human capital refers to the stock of knowledge, skills, and behavioral attributes that individuals accumulate and can translate into future productivity and well-being (Yueh, 2006; Qi et al., 2024). Following a competency-based framework, this study focuses on children’s learning outcomes and socio-emotional development.

Cognitive ability is measured using the composite cognitive test score provided in the CEPS baseline survey, which reflects students’ academic achievement and learning capacity. Non-cognitive ability captures students’ socio-emotional traits and behavioral characteristics. As the CEPS does not include a full-scale psychometric instrument for non-cognitive skills, this study constructs non-cognitive measures based on survey items that correspond to the “Big Five” personality framework. Following previous studies that have used CEPS data (Xu et al., 2018; Zhang et al., 2019; Liu et al., 2024), this study focuses on three dimensions with reliable measurement properties and sufficient data coverage: emotional stability, conscientiousness, and agreeableness.

With regard to the above three dimensions, emotional stability reflects students’ emotional regulation and stress management, and conscientiousness captures persistence, self-discipline, and task commitment. Finally, agreeableness reflects prosocial behavior and peer cooperation. Each dimension is constructed by averaging responses to multiple questionnaire items; higher values indicate stronger non-cognitive traits. These three dimensions are analyzed separately in the empirical analysis.

#### 3.2.2 Independent variables

The core explanatory variables capture different dimensions of parental involvement and parent–child relationships. Three indicators are now constructed based on students’ responses to the CEPS questionnaire.

Communication frequency measures how often parents discuss school-related issues and emotional experiences with their children. These issues include school life, peer relationships, interactions with teachers, emotions, and personal concerns, with responses coded on a 3-point scale (1 = never, 2 = sometimes, and 3 = often). The average score across the items is used, with higher values indicating more frequent communication.

Activity frequency refers to the frequency of joint activities between parents and children. Examples include having dinner together, reading, watching television, exercising, and participating in cultural or recreational activities. Responses range from 1 (never) to 6 (more than once a week), with the average score used to represent the intensity of parental companionship.

Closeness refers to the perceived emotional closeness between children and their parents. Students rated their relationship on a 3-point scale (1 = not close, 2 = average, and 3 = very close). This variable is treated as a continuous indicator, with higher values representing stronger emotional bonds.

#### 3.2.3 Control variables

Following prior research, this study controls for a set of individual and family characteristics that may influence children’s development. Individual-level controls include student sex, hukou type, only-child status, and boarding status. Family-level controls include family economic status, parents’ highest education level, and parental relationship quality. All control variables are derived from the CEPS baseline questionnaire and are consistently included across regression models.

#### 3.2.4 Mechanism variables: learning engagement and confidence

Learning engagement is constructed using students’ self-reported responses to a set of items in the CEPS questionnaire that capture persistence and effort in school-related tasks. Specifically, the measure is based on three Grade 6 items asking whether students would (i) try their best to attend school even when feeling unwell, (ii) persist in completing homework they dislike, and (iii) persist in completing homework even when it is time-consuming (CEPS items A12-01 to A12-03).

Responses are coded on a four-point Likert scale, with higher values indicating stronger engagement. We construct a composite learning engagement index by taking the row mean of the three items and standardize the resulting measure to have mean zero and unit variance. The scale exhibits satisfactory internal consistency (Cronbach’s alpha = 0.73), indicating that the items capture a coherent dimension of learning-related behavioral engagement.

Children’s confidence is measured using students’ self-assessed confidence in their future, based on CEPS item C25 (“How confident are you about your future?”). Responses range from “not confident at all” to “very confident” on a four-point scale. This item captures students’ perceived self-efficacy and expectations regarding their own abilities and future prospects, which have been widely linked to socio-emotional development in the literature. The variable is standardized prior to analysis to facilitate interpretation and comparability across specifications.

## 4 Results

### 4.1 Descriptive analysis

Table 2 reports the descriptive statistics of the main variables used in the empirical analysis, including measures of children’s cognitive ability, non-cognitive ability, parental involvement, and parent–child relationship quality.

**Table 2:** Descriptive statistics of key variables.

Variables	Obs.	Mean	Std. Dev.	Min	Max
Cognitive ability	19,487	9.939	3.759	0	22
Emotional stability	19,091	3.91	0.822	1	5
Conscientiousness	18,893	3.212	0.535	1	4
Agreeableness	19,238	2.331	0.885	1	4
Communication frequency	19,417	2.006	0.52	1	3
Activity frequency	19,345	3.689	1.068	1	6
Closeness	19,427	2.77	0.446	1	3

All variables are constructed from the CEPS 2013–2014 baseline student questionnaire. Differences in the number of observations across variables are due to item-level missingness.

The average cognitive ability score is 9.94, with a standard deviation of 3.76. These findings indicate substantial variation in academic performance among students. Regarding non-cognitive outcomes, emotional stability exhibits a relatively high mean value (3.91 on a 5-point scale). Conscientiousness and agreeableness average 3.21 and 2.33, respectively, on 4-point scales. These patterns suggest moderate to high levels of socio-emotional development, accompanied by meaningful individual differences.

Parental involvement variables display notable variations. The mean communication frequency is approximately 2.01 on a 3-point scale, suggesting that, for most students, parent–child communication occurs somewhere between “sometimes” and “often.” The average activity frequency is 3.69 on a 6-point scale, indicating that, while shared parent–child activities are present, for many families, they are not frequent on a weekly basis. Emotional closeness between parents and children has a relatively high mean (2.77 on a 3-point scale), implying that most students perceive having a generally close relationship with their parents.

The descriptive statistics reveal considerable heterogeneity in both children’s developmental outcomes and parental involvement patterns. The variations provide sufficient empirical basis for examining how different dimensions of parent–child relationships are associated with children’s cognitive and non-cognitive abilities in the subsequent regression analyses.

### 4.2 Basic measurement results

Building on the human capital production framework, a large body of literature has documented the important role of parental input in shaping children’s cognitive and non-cognitive development (Kuziemko, 2014). Existing studies have frequently emphasized parental investment in general. However, empirical evidence distinguishing specific forms of parental involvement—such as communication, shared activities, and emotional closeness—remains limited. This study seeks to fill this gap in research by examining how different dimensions of parent–child relationships are associated with children’s developmental outcomes.

Following Li et al. (2023) and Du et al. (2024), the baseline model specified in Equation (1) is now estimated. Here children’s cognitive and non-cognitive abilities are regressed on measures of parental involvement, along with a set of individual- and family-level control variables. The error term captures unobserved factors that may affect children’s human capital, including individual endowments and measurement errors.

(1) $$C_{h}=R_{h}\alpha_{h}+\beta_{h}X_{h}(I,F)+\varepsilon_{h}$$

In Equation (1), $C_{h}\;$ represents the cognitive and non-cognitive abilities within children’s human capital, and h denotes the regression sample; $R_{i,h}$ indicates the type of parent–child relationship. Then, $X_{h}(I,F)$ comprises the control variables, which include observable variables related to individuals (I) and families (F). Finally, $\varepsilon_{h}$ represents the random disturbance term, encompassing random shocks that are not controlled by parents but which may influence children’s human capital, as well as some omitted variables (such as unobserved innate abilities of children) and measurement errors.

Table 3 reports the baseline regression results for cognitive ability, where Columns (1) and (2) present estimates without and with control variables, respectively. The results show that both communication frequency and activity frequency are significantly and positively associated with children’s cognitive ability. Although the coefficients decline after controlling for individual and family characteristics, they remain statistically significant, indicating robust associations. In contrast, once controls are included, emotional closeness does not exhibit a significant relationship with cognitive performance. These findings suggest that cognitively-oriented parental inputs, such as frequent communication and shared activities, play a more prominent role in shaping academic outcomes.

**Table 3:** Baseline regression results: parental involvement and children’s abilities.

Variables	Cognitive ability (1)	Cognitive ability (2)
Communication	0.445***	0.373***
	(−0.059)	(−0.059)
Activity	0.706***	0.358***
	(−0.027)	(−0.029)
Closeness	−0.061	−0.000
	(−0.065)	(−0.064)
Controls	No	Yes
Observations	19,314	18,798
R-squared	0.053	0.118

***, **, and * represent the 1, 5, and 10% significance levels, respectively; standard errors are in parentheses.

To address concerns that a composite non-cognitive index may mask heterogeneous effects across dimensions, this study further estimates separate regressions for emotional stability, conscientiousness, and agreeableness. The results are reported in Table 4. Both before and after controlling for individual and family characteristics, across all three dimensions, communication frequency, activity frequency, and emotional closeness are positively and significantly associated with non-cognitive outcomes. However, the magnitude of each of these effects varies by trait. For example, emotional closeness exhibits a particularly strong association with emotional stability, while communication frequency plays a more pronounced role in conscientiousness and agreeableness. These results underscore the multidimensional nature of non-cognitive development; the results also highlight that different forms of parental involvement influence distinct socio-emotional traits.

**Table 4:** Effects of parental involvement on different dimensions of non-cognitive abilities.

Variables	Emotional stability		Conscientiousness		Agreeableness	
	(1)	(2)	(1)	(2)	(1)	(2)
Communication	0.127***	0.126***	0.152***	0.146***	0.334***	0.320***
	(−0.013)	(−0.014)	(−0.009)	(−0.009)	(−0.014)	(−0.014)
Activity	0.096***	0.086***	0.044***	0.041***	0.114***	0.099***
	(−0.006)	(−0.007)	(−0.004)	(−0.005)	(−0.007)	(−0.007)
Closeness	0.291***	0.268***	0.073***	0.063***	0.129***	0.126***
	(−0.016)	(−0.017)	(−0.01)	(−0.01)	(−0.015)	(−0.016)
Controls	No	Yes	No	Yes	No	Yes
Observations	18,970	18,526	18,771	18,334	19,120	18,623
R-squared	0.075	0.085	0.054	0.057	0.098	0.102

Where indicated, all regressions control for student sex, hukou type, boarding status, only-child status, family economic status, parents’ highest education level, and parental relationship quality.

The baseline results indicate that parental involvement is strongly associated with children’s development, albeit through different channels. Specifically, communication and shared activities are more closely linked to cognitive achievement, whereas emotional closeness is especially important for socio-emotional development. Together, Tables 3, 4 provide a comprehensive and nuanced picture of how parent–child relationships contribute to children’s human capital formation.

### 4.3 Robustness test

#### 4.3.1 Quantile regression analysis for cognitive ability

To examine the robustness of the baseline findings and to explore the distributional differences in the effects of parental involvement, this study now estimates quantile regressions of children’s cognitive ability at the 10th, 25th, 50th, 75th, and 90th percentiles. Bootstrap standard errors are employed to ensure reliable inference. Reported in Table 5, the results are largely consistent with the OLS benchmark, thereby confirming the robustness of the main conclusions.

**Table 5:** Robustness test: quantile regressions (bootstrap SEs).

Variables	Q-10	Q-25	Q-50	Q-75	Q-90
Communication frequency	0.369*** (0.089)	0.386*** (0.088)	0.333*** (0.079)	0.396*** (0.076)	0.271** (0.120)
Activity frequency	0.334*** (0.048)	0.365*** (0.043)	0.417*** (0.038)	0.372*** (0.042)	0.347*** (0.063)
Closeness	−0.061 (0.090)	−0.019 (0.103)	−0.050 (0.081)	0.035 (0.101)	0.203* (0.123)
Controls	Yes	Yes	Yes	Yes	Yes
Observations	18,798	18,798	18,798	18,798	18,798
Pseudo R-squared	0.049	0.059	0.06	0.065	0.054

Coefficients are reported with bootstrap standard errors in parentheses (500 replications). All specifications include the same set of individual and family control variables as in Table 3; ***, **, and * denote statistical significance at the 1, 5, and 10% levels, respectively.

Across all quantiles, both parental communication frequency and joint activity frequency are positively associated with children’s cognitive ability. The effect of joint activities is particularly stable and statistically significant throughout the entire distribution. This indicates that shared parent–child activities similarly benefit students with low, middle, and high levels of cognitive performance. Communication frequency also exhibits a consistently positive and significant effect, although the magnitude of the effect is relatively larger at the lower and middle quantiles and becomes smaller at the upper tail of the distribution. This pattern suggests diminishing marginal returns to parental communication among top-performing students.

In contrast, emotional closeness shows weaker robustness across quantiles. The coefficient of emotional closeness is not statistically significant for most parts of the distribution and becomes weakly significant at the 90th percentile. This finding suggests that emotional closeness may play a more limited role in shaping cognitive outcomes and may also be more relevant for students at the upper end of the performance distribution. The corresponding estimates are reported in Table 5.

#### 4.3.2 Quantile regression analysis for non-cognitive abilities

Reported in Table 6, the quantile regression results for non-cognitive outcomes further support the robustness of the baseline findings, while also revealing meaningful distributional heterogeneity across different dimensions. For emotional stability and conscientiousness, parental communication, joint activities, and emotional closeness exhibit positive and statistically significant effects across most quantiles, indicating broadly stable associations throughout the outcome distributions. In contrast, the effects on agreeableness are concentrated in the middle and upper parts of the distribution, while effects at the lower tail are limited, reflecting the more discrete distributional structure of this trait. Taken together, these results suggest that parental involvement consistently promotes children’s socio-emotional development, although the strength and distribution of effects vary across specific non-cognitive dimensions.

**Table 6:** A quantile regression analysis for non-cognitive abilities.

Variables	Panel A. Emotional Stability					Panel B. Conscientiousness					Panel C. Agreeableness				
	Q10	Q25	Q50	Q75	Q90	Q10	Q25	Q50	Q75	Q90	Q10	Q25	Q50	Q75	Q90
Communication	0.198***	0.181***	0.153***	0.122***	0.043***	0.185***	0.184***	0.183***	0.141***	0.128***	0	0.327***	0.440***	−0.000	0.382***
	−0.032	−0.02	−0.017	−0.016	−0.015	−0.018	−0.012	−0.011	−0.008	−0.01	(−)	−0.02	−0.02	(−)	−0.033
Activity	0.101***	0.091***	0.080***	0.092***	0.031***	0.050***	0.050***	0.040***	0.038***	0.038***	0	0.107***	0.133***	−0.000	0.078***
	−0.016	−0.011	−0.008	−0.009	−0.009	−0.009	−0.007	−0.005	−0.004	−0.005	(−)	−0.01	−0.01	(−)	−0.016
Closeness	0.480***	0.352***	0.259***	0.218***	0.152***	0.075***	0.074***	0.069***	0.059***	0.036***	0	0.319***	0.106***	−0.000	0.047
	−0.049	−0.025	−0.018	−0.019	−0.029	−0.019	−0.014	−0.012	−0.01	−0.013	(−)	−0.022	−0.021	(−)	−0.036
Controls	Yes	Yes	Yes	Yes	Yes	Yes	Yes	Yes	Yes	Yes	Yes	Yes	Yes	Yes	Yes
Observations	18,526	18,526	18,526	18,526	18,526	18,334	18,334	18,334	18,334	18,334	18,623	18,623	18,623	18,623	18,623

This table reports quantile regression estimates for emotional stability (Panel A), conscientiousness (Panel B), and agreeableness (Panel C) at the 10th, 25th, 50th, 75th, and 90th percentiles. Robust standard errors are reported in parentheses. All specifications include the same set of individual and family control variables as in the baseline regressions (not reported). For agreeableness, coefficients reported as zero with missing standard errors indicate non-identification at that quantile due to the discrete outcome distribution. ***, **, and * denote statistical significance at the 1, 5, and 10% levels, respectively.

### 4.4 Mechanism analysis: how parental involvement affects children’s cognitive and non-cognitive abilities

To clarify the mechanisms through which parental involvement influences children’s cognitive and non-cognitive abilities, this study conducts a mediation analysis focusing on two theoretically grounded and empirically observable transmission channels: children’s learning engagement and confidence. Learning engagement and confidence are measured using the CEPS survey items described in Section 3 and capture, respectively, children’s behavioral investment in learning and their perceived self-efficacy regarding future outcomes.

#### 4.4.1 Learning engagement as a mediating mechanism

Following standard mediation frameworks, we first estimate the total effect of parental involvement on children’s cognitive ability. We then examine the effect of parental involvement on learning engagement and finally include both parental involvement and learning engagement in the cognitive ability regression. The statistical significance of the indirect effect is assessed using a nonparametric bootstrap procedure with 1,000 replications.

The results in provide clear evidence of a partial mediating role of learning engagement. As reported in Table 7 panel A, the estimated indirect effect (a × b) equals 0.049 (*p* < 0.001), with a 95% bootstrap confidence interval of [0.039, 0.059], which excludes zero. In proportional terms, learning engagement accounts for approximately 6.1% of the total effect of parental involvement on cognitive ability. This suggests that parental involvement enhances children’s cognitive performance partly by fostering greater persistence and effort in learning activities, while a substantial direct effect remains.

**Table 7:** Mechanism analysis: mediating channels of parental involvement.

Outcome variable	Indirect effect (ab)	95% CI for ab	Mediated share
Panel A. Learning engagement as a mediating mechanism			
Cognitive ability	0.049***	[0.039, 0.059]	0.061
Emotional stability	0.014***	[0.011, 0.016]	0.064
Conscientiousness	0.066***	[0.060, 0.072]	0.566
Agreeableness	0.019***	[0.016, 0.021]	0.07
Panel B. Confidence as a mediating mechanism			
Emotional stability	0.054***	[0.049, 0.059]	0.256
Conscientiousness	0.029***	[0.026, 0.032]	0.25
Agreeableness	0.053***	[0.048, 0.057]	0.199

This table reports bootstrap mediation results examining the mechanisms through which parental involvement affects children’s cognitive and non-cognitive abilities. Indirect effects (ab) are estimated using 1,000 bootstrap replications. The mediated share is calculated as the ratio of the indirect effect to the total effect. All models include the same set of control variables as in the baseline regressions. ***p < 0.01, **p < 0.05, *p < 0.10.

Turning to non-cognitive outcomes, the mediating role of learning engagement is more pronounced and varies across dimensions. The indirect effect is particularly strong for conscientiousness, where learning engagement explains over half of the total effect of parental involvement. By contrast, the mediated shares for emotional stability and agreeableness are positive but more modest. These findings indicate that learning-related behavioral investment is especially relevant for non-cognitive traits closely associated with diligence, responsibility, and self-discipline.

#### 4.4.2 Confidence as a complementary mechanism for non-cognitive development

To further explore the mechanisms underlying children’s non-cognitive development, we examine confidence as an additional mediator. Confidence captures students’ beliefs in their own abilities and future prospects and reflects an important psychological channel through which parental involvement may shape socio-emotional outcomes.

Bootstrap mediation results reported in Table 7 panel B show that confidence significantly mediates the relationship between parental involvement and all three non-cognitive dimensions. The indirect effects account for approximately 20–25% of the total effects, underscoring the importance of psychological pathways alongside behavioral investment. Taken together, these results suggest that parental involvement promotes non-cognitive development through both learning-related behaviors and students’ confidence in their own capabilities.

### 4.5 Further evidence: heterogeneity and contextual conditions

This section provides further evidence on whether the effects of parental involvement on children’s cognitive and non-cognitive abilities vary systematically across individual characteristics and family contexts. Rather than interpreting such variations as transmission mechanisms, the analyses in this section focus on heterogeneity and contextual conditions under which parental involvement operates. Two sets of analyses are conducted. The first examines heterogeneity by children’s demographic and institutional characteristics, while the second investigates how the family expectation environment conditions the effectiveness of parental involvement.

#### 4.5.1 Heterogeneity by gender, hukou status, and boarding status

While subsample regressions suggest potentially heterogeneous patterns across groups, direct comparisons of coefficients across subsamples do not constitute formal statistical tests. To address this limitation, this study adopts an interaction-based regression framework in the full sample. Specifically, interaction terms between parental involvement measures and indicators for gender, household registration (hukou), and boarding status are introduced into the baseline regression model. Wald tests are then applied to assess whether the interaction terms are jointly significant.

##### 4.5.1.1 Heterogeneity analysis for cognitive ability

As reported in Table 8, clear evidence of gender heterogeneity emerges for cognitive ability. The interaction between activity frequency and the male indicator is significantly negative, whereas the interaction between parent–child emotional closeness and the male indicator is significantly positive. Moreover, the joint Wald test strongly rejects the null hypothesis that all gender-related interaction terms equal zero (*p* < 0.001). Substantively, these results suggest that shared parental activities are more strongly associated with cognitive outcomes for girls, while emotional closeness plays a comparatively more important role for boys.

**Table 8:** Heterogeneity analysis for cognitive ability.

Variables	Gender (Male = 1)	Hukou (Non-agric. = 1)	Boarding (Yes = 1)
Communication × group	−0.212* (0.116)	−0.103 (0.117)	−0.191 (0.122)
Activity × group	−0.221*** (0.053)	−0.019 (0.056)	0.019 (0.059)
Closeness × group	0.276** (0.127)	0.225* (0.130)	0.132 (0.134)
Communication	0.484*** (0.085)	0.430*** (0.090)	0.436*** (0.072)
Activity	0.478*** (0.040)	0.369*** (0.044)	0.351*** (0.035)
Closeness	−0.160* (0.093)	−0.128 (0.101)	−0.043 (0.079)
Controls	Yes	Yes	Yes
Observations	18,798	18,798	18,798
R-squared	0.119	0.118	0.118
Joint test of interactions (*p*-value)	0	0.362	0.427

Robust standard errors are in parentheses. The joint test reports Wald tests for the null hypothesis that all interaction coefficients equal 0; ***p < 0.01, **p < 0.05, and *p < 0.10.

In contrast, no robust heterogeneity is detected with respect to hukou status or boarding status. Although the interaction between emotional closeness and hukou status is marginally significant, the joint Wald tests fail to reject the null hypothesis of homogeneous effects across these dimensions. These findings indicate that observed differences in subsample regressions primarily reflect level differences rather than statistically significant differences in marginal effects.

##### 4.5.1.2 Heterogeneity analysis for non-cognitive abilities

Using the same interaction-based framework, Table 9A reports heterogeneity results for non-cognitive outcomes, including emotional stability, conscientiousness, and agreeableness. Compared with cognitive ability, heterogeneity patterns for non-cognitive traits are present but less systematic.

**Table 9:** Heterogeneity analysis for non-cognitive abilities.

Variables	Panel A. Dependent variables: Emotional Stability			Panel B. Dependent variables: Conscientiousness			Panel C. Dependent variables: Agreeableness		
	(1) Gender	(2) Hukou	(3) Boarding	(4) Gender	(5) Hukou	(6) Boarding	(7) Gender	(8) Hukou	(9) Boarding
Communication	0.159***	0.105***	0.128***	0.143***	0.124***	0.139***	0.345***	0.305***	0.327***
	(−0.019)	(−0.022)	(−0.017)	(−0.012)	(−0.015)	(−0.012)	(−0.02)	(−0.022)	(−0.017)
Communication × Group	−0.059**	−0.047*	−0.010	0.006	0.038**	0.02	0.028	−0.005	−0.021
	(−0.027)	(−0.028)	(−0.028)	(−0.018)	(−0.019)	(−0.018)	(−0.029)	(−0.014)	(−0.03)
Activity	0.090***	0.103***	0.097***	0.037***	0.044***	0.043***	0.104***	0.102***	0.101***
	(−0.009)	(−0.011)	(−0.009)	(−0.006)	(−0.007)	(−0.006)	(−0.01)	(−0.011)	(−0.009)
Activity × Group	−0.007	−0.011	−0.033**	0.008	−0.005	−0.007	−0.005	−0.005	−0.007
	(−0.012)	(−0.013)	(−0.013)	(−0.008)	(−0.009)	(−0.009)	(−0.014)	(−0.014)	(−0.015)
Closeness	0.226***	0.287***	0.298***	0.053***	0.098***	0.059***	0.141***	0.163***	0.148***
	(−0.023)	(−0.027)	(−0.021)	(−0.014)	(−0.016)	(−0.012)	(−0.023)	(−0.024)	(−0.019)
Closeness × Group	0.074**	−0.028	−0.100***	0.018	−0.061***	0.013	−0.066**	−0.066**	−0.074**
	(−0.033)	(−0.031)	(−0.034)	(−0.019)	(−0.02)	(−0.02)	(−0.032)	(−0.032)	(−0.034)
Observations	18,526	18,526	18,526	18,334	18,334	18,334	18,623	18,623	18,623
R-squared	0.086	0.086	0.087	0.057	0.058	0.057	0.103	0.102	0.103
Joint test (*p*-value)	0.03	0.081	0	0.329	0.012	0.52	0.031	0.195	0.031

All regressions control for the same set of individual and family covariates as in the baseline specifications. “Group” refers to gender, hukou status, or boarding status, depending on the column. Robust standard errors are reported in parentheses. “Joint test” reports the *p*-value of a Wald test for the null hypothesis that all three interaction terms equal zero. ***, **, and * denote statistical significance at the 1, 5, and 10% levels, respectively.

For emotional stability, several interaction terms are statistically significant, and the joint Wald tests reject the null hypothesis of homogeneous effects along gender and boarding dimensions. These results suggest that the socio-emotional returns to parental involvement vary across children’s demographic and institutional contexts. For conscientiousness and agreeableness, heterogeneity effects are more limited. Although some individual interaction terms—particularly those involving emotional closeness—are statistically significant, the joint Wald tests indicate that such heterogeneity is not consistently present across all dimensions of parental involvement.

#### 4.5.2 Family expectation environment as a contextual condition

Beyond demographic characteristics, parental involvement may also operate differently depending on the broader family expectation environment. To examine this possibility, this study focuses on two conceptually distinct dimensions of parental educational orientation: parental educational aspirations and parental academic pressure.

Parental educational aspiration (B31) is measured by the question “What is the highest level of education your parents expect you to receive?” and captures parents’ long-term, forward-looking goals regarding children’s educational attainment. In contrast, parental academic requirement (B30), measured by the question “What is your parents’ requirement on your academic record?,” reflects the intensity of short-term performance pressure experienced by children.

Existing research suggests that long-term aspirations and short-term academic pressure may condition how parental involvement translates into children’s developmental outcomes through different channels (Hill and Tyson, 2009; Wang and Sheikh-Khalil, 2014). Educational aspirations may provide a goal-oriented framework that enhances the effectiveness of emotionally supportive involvement, whereas excessive academic pressure may alter the emotional climate of parent–child interactions and weaken socio-emotional well-being.

##### 4.5.2.1 Expectation-conditioned effects on cognitive outcomes

Panel A of Table 10 reports the interaction results for cognitive ability. A clear and robust pattern emerges for parent–child emotional closeness. The interaction between emotional closeness and parental educational aspiration is positive and statistically significant (*β* = 0.209, *p* < 0.01), while the corresponding interaction with parental academic requirement is statistically insignificant.

**Table 10:** Moderating effects of parental educational aspirations and academic pressure on the relationship between parental involvement and children’s development.

Variables	(1) Moderator = z(B31)	(2) Moderator = z(B30)
Panel A. Dependent variable: Cognitive ability		
Communication	0.237*** (0.056)	0.174*** (0.056)
Activity	0.260*** (0.028)	0.285*** (0.028)
Closeness	−0.097 (0.063)	−0.141** (0.062)
Moderator	0.070 (0.149)	−0.641*** (0.159)
Communication × Moderator	0.029 (0.053)	0.077 (0.056)
Activity × Moderator	0.000 (0.025)	−0.027 (0.026)
Closeness × Moderator	0.209*** (0.056)	−0.068 (0.059)
Controls	Yes	Yes
Observations	18,699	18,720
R-squared	0.193	0.201
Joint test of interactions (*p*-value)	0	0.382

Robust standard errors are reported in parentheses. Parental educational aspiration (B31) and parental academic requirement (B30) are standardized (z-scores) and enter the regressions both directly and through interaction terms with parental involvement measures. All specifications include the same set of individual and family control variables as in the baseline regressions. “Joint test of interactions” reports the *p*-value of a Wald test for the null hypothesis that all interaction coefficients equal zero. ***, **, and * denote statistical significance at the 1, 5, and 10% levels, respectively.

**Figure 1:**
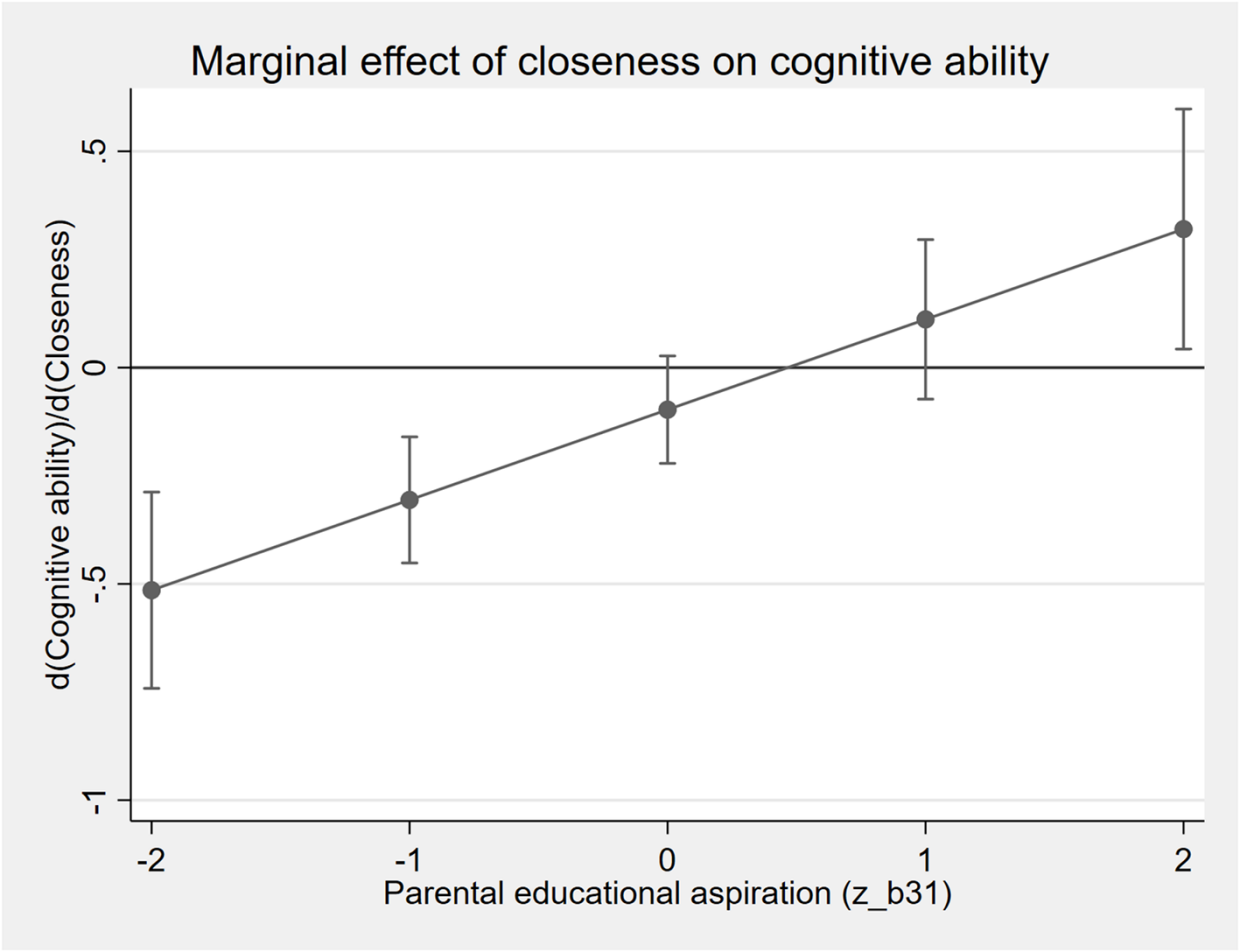
Marginal effect of emotional closeness on cognitive ability across levels of parental educational aspiration.

Figure 1 illustrates this pattern by plotting the marginal effect of emotional closeness across different levels of parental educational aspiration. When parental aspiration is low, emotional closeness is associated with a negative or insignificant effect on cognitive ability. As parental aspiration increases, the marginal effect becomes positive and steadily strengthens. This finding suggests that emotional closeness enhances children’s cognitive performance primarily when embedded within a strong long-term educational orientation. In contrast, the interaction terms involving communication and activity frequency are not statistically significant, indicating that their cognitive returns are relatively stable across different expectation environments.

##### 4.5.2.2 Expectation-conditioned effects on socio-emotional outcomes

**Figure 2:**
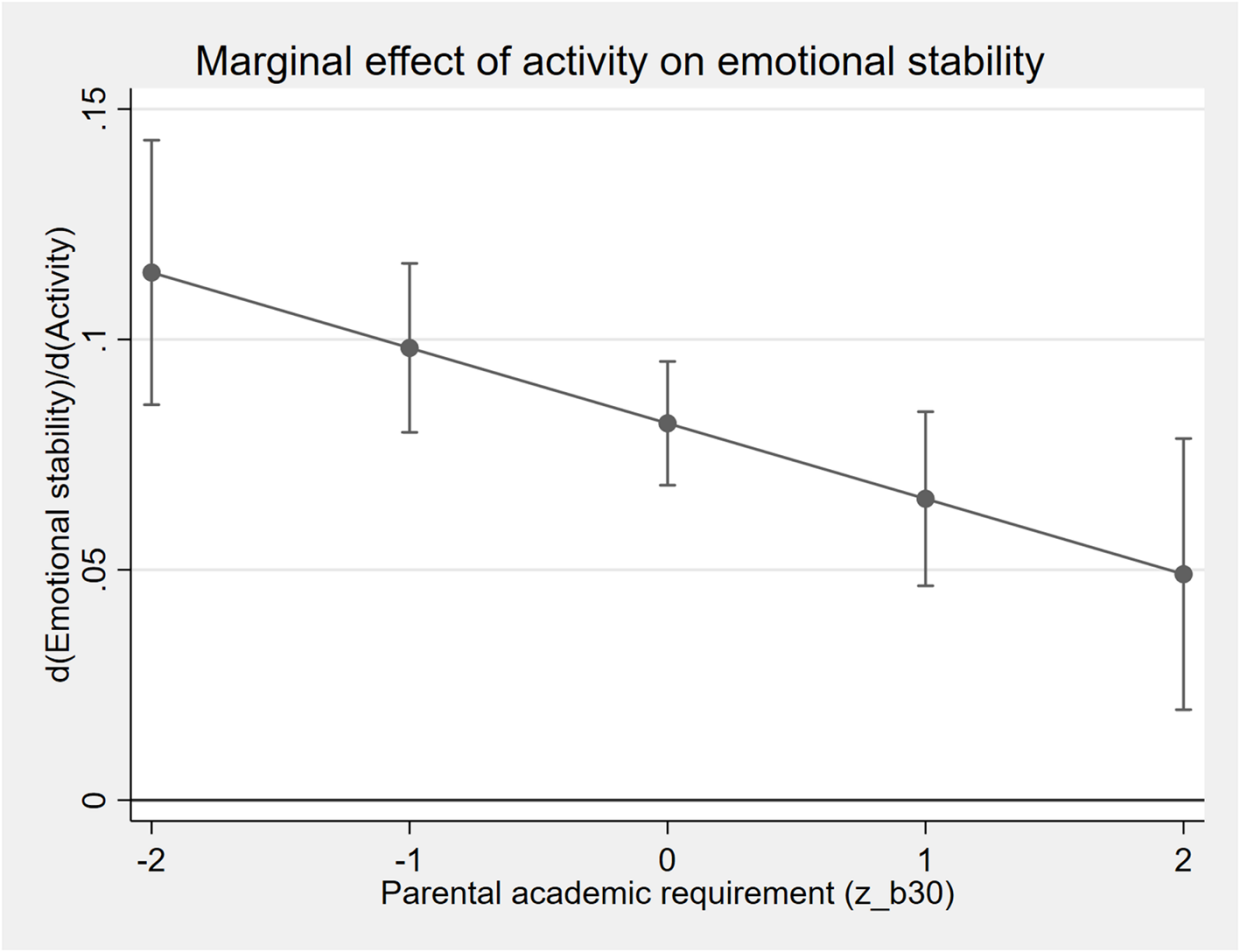
Marginal effect of parental activity frequency on emotional stability by parental academic requirement.

Panels B–D of Table 11 report the interaction results for non-cognitive outcomes. Among the three dimensions, emotional stability exhibits the most pronounced expectation-conditioned pattern. Specifically, the interaction between activity frequency and parental academic requirement is negative and statistically significant (*β* = −0.016, *p* < 0.05). Figure 2 visualizes this relationship by plotting the marginal effect of parental activity on emotional stability across different levels of academic pressure. When parental academic pressure is low, shared activities are strongly associated with higher emotional stability. As pressure increases, the emotional benefits of such activities decline substantially.

**Table 11:** Moderating effects of parental educational aspirations and academic pressure on non-cognitive abilities.

Variables	Panel B. Dependent variable: Emotional stability		Panel C. Dependent variable: Conscientiousness		Panel D. Dependent variable: Agreeableness	
	(1) B31	(2) B30	(1) B31	(2) B30	(1) B31	(2) B30
Communication	0.122*** (0.014)	0.123*** (0.014)	0.137*** (0.009)	0.130*** (0.009)	0.305*** (0.014)	0.296*** (0.014)
Activity	0.079*** (0.007)	0.082*** (0.007)	0.034*** (0.005)	0.036*** (0.005)	0.090*** (0.007)	0.094*** (0.007)
Closeness	0.263*** (0.017)	0.262*** (0.017)	0.057*** (0.010)	0.053*** (0.010)	0.118*** (0.016)	0.113*** (0.016)
Moderator	−0.118*** (0.043)	0.091** (0.045)	0.006 (0.025)	−0.093*** (0.026)	−0.070* (0.041)	−0.037 (0.041)
Communication × Moderator	0.030** (0.014)	−0.015 (0.014)	−0.002 (0.009)	0.013 (0.009)	0.035** (0.015)	−0.013 (0.014)
Activity × Moderator	0.011 (0.007)	−0.016** (0.007)	0.003 (0.004)	0.005 (0.004)	0.009 (0.007)	−0.011* (0.007)
Closeness × Moderator	0.012 (0.016)	−0.006 (0.016)	0.015 (0.009)	−0.005 (0.010)	0.013 (0.015)	0.000 (0.015)
Controls	Yes	Yes	Yes	Yes	Yes	Yes
Observations	18,428	18,451	18,244	18,259	18,529	18,547
R-squared	0.091	0.091	0.073	0.076	0.113	0.12
Joint test of interactions (*p*-value)	0.002	0.004	0.244	0.142	0.001	0.097

Robust standard errors are reported in parentheses. Parental educational aspiration (B31) and parental academic requirement (B30) are standardized (z-scores). Each column reports interaction-based regressions that examine whether the associations between parental involvement and children’s non-cognitive abilities vary across parental expectation environments. All regressions control for the same set of individual and family covariates as in the baseline specifications. “Joint test” reports the *p*-value of a Wald test for the null hypothesis that all interaction terms equal zero. ***, **, and * denote statistical significance at the 1, 5, and 10% levels, respectively.

For conscientiousness and agreeableness, most interaction terms are small and statistically insignificant. These results indicate limited evidence that parental expectations systematically condition the effects of parental involvement on these traits.

#### 4.5.3 Synthesis of heterogeneity and moderation results

Taken together, the results in this section indicate that the effects of parental involvement are not uniform across children or family environments. Demographic heterogeneity is most pronounced for cognitive ability, particularly along the gender dimension. In addition, the family expectation environment plays an important contextual role: long-term educational aspirations amplify the cognitive returns of emotionally supportive involvement, whereas high short-term academic pressure weakens the socio-emotional benefits of parental engagement, especially for emotional stability.

Importantly, these findings highlight that parental involvement does not operate in isolation. Its effectiveness depends on both children’s characteristics and the broader expectation context within the family. Rather than constituting distinct mechanisms, parental aspirations and academic pressure shape the conditions under which parental involvement yields stronger or weaker developmental returns.

## 5 Conclusion and discussions

Using nationally-representative CEPS data, this study investigates how parental involvement shapes children’s cognitive and non-cognitive development in China. By integrating baseline regressions, robustness checks, heterogeneity analysis, and mechanism tests, the results provide a comprehensive assessment of the role of parent–child interactions in children’s human capital formation.

The empirical results consistently show that parental involvement—measured by communication frequency, shared activities, and emotional closeness—is positively associated with children’s cognitive ability and multiple dimensions of non-cognitive development. These findings remain robust across alternative model specifications and quantile regressions, which in turn indicates that the results are not driven by specific estimation choices or distributional assumptions. Overall, active parental engagement emerges as a stable and meaningful predictor of children’s developmental outcomes.

The study’s heterogeneity analysis further reveals that the effects of parental involvement vary across groups; gender represents the most salient dimension of heterogeneity. Formal interaction-based tests indicate that male children and female children respond differently to specific forms of parental involvement, particularly shared activities and emotional closeness. In contrast, while descriptive differences are observed by household registration and boarding status, the corresponding interaction terms are not jointly significant. This finding suggests that disparities across these groups primarily reflect differences in baseline conditions and access to parental involvement, rather than statistically-distinct marginal effects.

Beyond documenting average associations and heterogeneous effects, the mechanism analysis sheds light on how parental involvement translates into children’s developmental outcomes. The mediation results indicate that learning engagement constitutes an important behavioral channel linking parental involvement to cognitive ability. Parental communication, shared activities, and supervision enhance children’s cognitive performance partly by fostering greater effort, persistence, and engagement in learning tasks. Although the mediating share is modest, the results point to partial mediation, highlighting that parental involvement affects cognitive development through both behavioral investment and other complementary channels.

For non-cognitive development, the mechanisms are more differentiated across dimensions. Learning engagement plays a particularly prominent mediating role for conscientiousness, consistent with its close conceptual link to persistence, responsibility, and self-discipline. In contrast, emotional stability and agreeableness are influenced through both behavioral and psychological pathways. Mediation results further show that children’s confidence in their future significantly transmits the effects of parental involvement to all three non-cognitive outcomes, underscoring the importance of self-efficacy and expectations as key socio-emotional mechanisms. Taken together, these findings suggest that parental involvement operates through a combination of behavioral and psychological channels, rather than through a single unified pathway.

A key contribution of this study lies in situating these findings within a broader cross-cultural perspective. While international research has widely documented the benefits of parental involvement, the dominant pathways differ across institutional and cultural settings. In many Western societies, parenting practices emphasize autonomy, emotional expression, and child-centered interaction, with relatively greater attention to non-cognitive outcomes. In contrast, Chinese parenting is shaped by Confucian family norms, strong academic expectations, and an examination-oriented education system. These features tend to channel parental involvement toward learning-related communication, academic supervision, and structured activities, which may explain why cognitive outcomes respond particularly strongly to parental engagement in the Chinese context.

Institutional factors, such as the household registration system and boarding school arrangements, further distinguish China from many Western settings. The structural constraints faced by rural families and boarding students limit opportunities for daily parent–child interaction, which may potentially amplify the importance of family-based inputs where such interaction is available. In this sense, the observed effects reflect both the universal mechanisms of parental influence and the context-specific amplifications that arise from China’s social and educational institutions.

From a policy perspective, the findings of this study highlight the importance of supportive parents in terms of providing effective, learning-oriented engagement, rather than focusing exclusively on academic outcomes. Family education programs that enhance parents’ communication skills and encourage shared educational activities may be particularly beneficial. This is especially true for families facing structural disadvantages. At the same time, greater attention should be paid to children’s non-cognitive development, which remains relatively undervalued in China’s exam-oriented system.

Several limitations of this study should be acknowledged. First, the measurement of non-cognitive abilities and mediating mechanisms is constrained by available CEPS items. Second, the observational nature of the data limits causal inference, despite extensive controls and robustness checks. Third, although a cross-cultural discussion is provided, this study does not conduct direct international comparisons. Future research using longitudinal or cross-national data could further assess the dynamics and generalizability of the mechanisms identified in this study.

Overall, this study demonstrates that, in China, parental involvement plays a critical role in shaping children’s cognitive and non-cognitive development. The results highlight behavioral pathways, gender-based heterogeneity, and culturally specific mechanisms, the findings contribute to a more nuanced understanding of how family processes influence children’s human capital formation in a specific institutional and cultural context.

## Data Availability

The original contributions presented in the study are included in the article/supplementary material, further inquiries can be directed to the corresponding authors.
